# Author Correction: Axion topology in photonic crystal domain walls

**DOI:** 10.1038/s41467-024-52438-8

**Published:** 2024-09-17

**Authors:** Chiara Devescovi, Antonio Morales-Pérez, Yoonseok Hwang, Mikel García-Díez, Iñigo Robredo, Juan Luis Mañes, Barry Bradlyn, Aitzol García-Etxarri, Maia G. Vergniory

**Affiliations:** 1 Institute for Theoretical Physics, ETH Zurich, 8093 Zürich, Switzerland; 2 Donostia International Physics Center, Paseo Manuel de Lardizabal 4, 20018 Donostia-San Sebastian, Spain; 3 Material and Applied Physics Department, University of the Basque Country (UPV/EHU), Donostia-San Sebastian, Spain; 4 Department of Physics, University of Illinois at Urbana-Champaign, Urbana, IL USA; 5 Physics Department, University of the Basque Country (UPV/EHU), Bilbao, Spain; 6 Max Planck Institute for Chemical Physics of Solids, D-01187 Dresden, Germany; 7 IKERBASQUE, Basque Foundation for Science, María Díaz de Haro 3, 48013 Bilbao, Spain

**Keywords:** Topological insulators

Correction to: *Nature Communications* 10.1038/s41467-024-50766-3, published online 09 August 2024

In this article, the details for reference 77 were incorrect.

Old reference:

Devescovi, C. et al. Tutorial 2.0: computing topological invariants in 3D photonic crystals. In *Opt. Mater. Express* (2024) 10.1364/OME.52906 (in press).

New reference:

Devescovi, C. et al. Tutorial 2.0: computing topological invariants in 3D photonic crystals. *Opt. Mater. Express*. **14**, 2161–2177. 10.1364/OME.529068 (2024).

The original article has been corrected.

