# Supplementary material for: Axion topology in photonic crystal domain walls
Source: Nat Commun. 2024 Aug 9;15:6814. doi: 10.1038/s41467-024-50766-3 (PMC11316128; doi:10.1038/s41467-024-50766-3)
Supplement: Supplementary file 1 — Supplementary Information [file 41467_2024_50766_MOESM1_ESM.pdf]

Supplementary Materials for "Axion Topology in Photonic Crystal Domain Walls"

Chiara Devescovi*,[1,2,*] Antonio Morales-Pérez*,[2,3,†] Yoonseok Hwang,[4] Mikel García-Díez,[2,5] Iñigo Robredo,[6,2] Barry Bradlyn,[4] Juan Luis Mañes,[5] Aitzol García-Etxarri,[2,7,‡] and Maia G. Vergniory[6,2,§]

[1] *Institute for Theoretical Physics, ETH Zurich, 8093 Zürich, Switzerland*
[2] *Donostia International Physics Center, Paseo Manuel de Lardizabal 4, 20018 Donostia-San Sebastian, Spain.*
[3] *Material and Applied Physics Department, University of the Basque Country (UPV/EHU), Donostia-San Sebastian, Spain.*
[4] *Department of Physics, University of Illinois at Urbana-Champaign, Urbana, IL, USA*
[5] *Physics Department, University of the Basque Country (UPV/EHU), Bilbao, Spain*
[6] *Max Planck Institute for Chemical Physics of Solids, Dresden D-01187, Germany*
[7] *IKERBASQUE, Basque Foundation for Science, María Díaz de Haro 3, 48013 Bilbao, Spain.*

(Dated: July 11, 2024)

I. MAGNETIC SPACE GROUP ANALYSIS

In this section, we analyze the Magnetic Space Group (MSG) for the gyrotropic Photonic Crystals (PhCs). The MSG assignation is conducted on the dielectric structure, via the use of the FINDSYM software [1]. We perform this analysis for the case of a Supercell Modulation (SM) of a $N = 3$ period, with phase $\phi = 0, \pi$ and in presence of an external magnetic field :

$$\mathbf{H} = (|h|\cos(\sigma), |h|\sin(\sigma), H_z) \tag{1}$$

with $|h| << |H_z|$. We evaluate the symmetry content of the electromagnetic fields, via the study of the transformation properties of the **D** electric and **B** magnetic modes supported by the PhC, using the MIT Photonic Bands (MPB) solver [2], as explained in the Method section.

We label the irreducible representations (irreps) at the high-symmetry points (HSPs) according to the notation of Bilbao Crystallographic Server (BCS) [3, 4]. We compute the symmetry vector $\tilde{\mathbf{v}}$, which contains the multiplicity of each irrep in the little group of each HSP. From these, we finally extract the corresponding Symmetry Indicators (SI) [5–10]. Please note that all operations and coordinates are expressed with respect to the following primitive lattice vectors:

$$\mathbf{e}_1 = \hat{\mathbf{x}}, \quad \mathbf{e}_2 = \hat{\mathbf{y}}, \quad \mathbf{e}_3 = 3\hat{\mathbf{z}}. \tag{2}$$

These lattice vectors are those of the BCS parent Space Group (SG) #224. We will stick to this cartesian system even for the lower symmetry phases.

A. Case with $|h| = 0$

When the gyrotropic axis is along z, i.e. in presence of an external $\mathbf{H} = (0, 0, H_z)$ magnetic field, the PhCs are in MSG #67.505. Please note that there is a $\pi/4$ rotation in the OXY plane between SG #224 and MSG #67.505.

To label the irreps at the HSP, we consider the maximal unitary subgroup of MSG #67.505 (Cm'm'a), i.e. Space Group (SG) #13 ($P2/m$), whose generators are:

$$\{C_{2z}|\frac{1}{2}, \frac{1}{2}, 0\}, \quad \{m_z|\frac{1}{2}, \frac{1}{2}, 0\}, \quad \{\bar{1}|0, 0, 0\}, \tag{3}$$

expressed in the basis of Eq. (2). The anti-unitary generators of MSG #67.505 are:

$$\begin{gathered}\{C'_{2_{110}}|\frac{1}{2}, \frac{1}{2}, 0\}, \quad \{m'_{110}|\frac{1}{2}, \frac{1}{2}, 0\}, \\ \{C'_{2_{1\bar{1}0}}|0, 0, 0\}, \quad \{m'_{1\bar{1}0}|0, 0, 0\},\end{gathered} \tag{4}$$

* cdevescovi@phys.ethz.ch
† antonio.morales@dipc.org
‡ aitzolgarcia@dipc.org
§ maia.vergniory@cpfs.mpg.de

which are also expressed in the basis of Eq. (2) and where the prime indicates the time-reversal operation. Please note that there is a $\pi/2$ rotation in the OYZ plane between MSG #67.505 and SG #13.

The symmetry vectors, computed for the lowest six transverse bands of the PhC are:

$$\begin{aligned}\tilde{\mathbf{v}}^T_{\phi=0} = [&3A_1, 3B_1, C_1^- + 2C_2^+ + 3C_2^-, 3D_1, 3E_1, (\blacksquare)^{2T} \\ &+2\Gamma_2^+ + 2\Gamma_2^-, Y_1^- + 2Y_2^+ + 3Y_2^-, 3Z_2^+ + 3Z_2^-]\end{aligned} \tag{5}$$

and

$$\begin{aligned}\tilde{\mathbf{v}}^T_{\phi=\pi} = [&3A_1, 3B_1, C_1^- + 2C_2^+ + 3C_2^-, 3D_1, 3E_1, (\blacksquare)^{2T} \\ &+2\Gamma_2^+ + 2\Gamma_2^-, Y_1^+ + 3Y_2^+ + 2Y_2^-, 3Z_2^+ + 3Z_2^-]\end{aligned} \tag{6}$$

where $(\blacksquare)^{2T}$ indicates the irregular symmetry content at Γ and $\omega = 0$ arising from transversality of the electromagnetic waves [11, 12], where $()^T$ labels the transverse bands and where $\tilde{}$ superscript indicates that the analysis is done after the introduction of the SM.

Note that HSPs labeled as Y, Z, C correspond to the following reduced coordinates in the Brillouin Zone (BZ), whose little group has solely 1-dimensional irreps:

$$Y = (\pi, \pi, 0), \quad Z = (0, 0, \pi), \quad C = (\pi, \pi, \pi). \tag{7}$$

The HSPs A, B, D, E, whose little group has a single 2-dimensional irrep, correspond to the following:

$$A = (\pi, 0, 0), \;\; B = (0, \pi, 0), \;\; D = (0, \pi, \pi), \;\; E = (\pi, 0, \pi). \tag{8}$$

On the other hand, for the lowest modes of the Tranversality-Enforced Tight-Bindind (TETB) model, we obtain:

$$\begin{aligned}\tilde{\mathbf{v}}^{T+L}_{\phi=0} =& [6A_1, 6B_1, 3C_1^+ + 4C_1^- + 2C_2^+ + 3C_2^-, 6D_1, \\ & 6E_1, 2\Gamma_1^+ + 4\Gamma_1^- + 2\Gamma_2^+ + 4\Gamma_2^-, 3Y_1^+ + 4Y_1^- \\ & + 2Y_2^+ + 3Y_2^-, 3Z_1^+ + 3Z_1^- + 3Z_2^+ + 3Z_2^-]\end{aligned} \tag{9}$$

and

$$\begin{aligned}\tilde{\mathbf{v}}^{T+L}_{\phi=\pi} =& [6A_1, 6B_1, 3C_1^+ + 4C_1^- + 2C_2^+ + 3C_2^-, 6D_1, \\ & 6E_1, 2\Gamma_1^+ + 4\Gamma_1^- + 2\Gamma_2^+ + 4\Gamma_2^-, 4Y_1^+ + 3Y_1^- \\ & + 3Y_2^+ + 2Y_2^-, 3Z_1^+ + 3Z_1^- + 3Z_2^+ + 3Z_2^-].\end{aligned} \tag{10}$$

These equations are in correspondence with Eqs. M6-M9 of the main text, where we take into account solely inversion symmetry.

After having identified the irregular irrep content at Γ, as $(\blacksquare)^{2T} = -\Gamma_1^+ + \Gamma_1^- + 2\Gamma_2^-$, consistent with symmetry-constrained decomposition for point group $2/m$ as in Refs. [11, 12], we can split the TETB symmetry vector as follows: $\tilde{\mathbf{v}}^{T+L}_{\phi} = \tilde{\mathbf{v}}^{T}_{\phi} + \tilde{\mathbf{v}}^{L}_{\phi}$.

Since $\tilde{\mathbf{v}}^{L}_{\phi}$ have trivial SI, we can extract the transverse SI for the photonic bands obtaining:

$$\begin{aligned}\nu^{T+L}_{\phi=0} &= \{1, 0\} \\ \nu^{T+L}_{\phi=\pi} &= \{1, 1\}\end{aligned} \tag{11}$$

corresponding to the $\mathbb{Z}_2 \times \mathbb{Z}_2$ magnetic SI group, see Table 5 in Ref. [5].

B. Case with $|h| << |H_z|$

When we apply a small off-z tilt to the external magnetic field, as described by Eq. (1), the PhC undergoes subduction to MSG #2.4 ($P\bar{1}$). As long as the $|h|$ perturbation is weak enough as compared to $|H_z|$, the transition occurs without closing the bulk Chern gap, leaving the Chern numbers unaffected, with

$$(C_x, C_y, C_z) = (0, 0, 1). \tag{12}$$

More precisely, the $|h|$ component introduces a small deviation from the Weyl folding commensurate condition, which results in a gradual shrinking of the bulk 3D Chern gap as $|h|$ is increased. However, as long as the bulk 3D Chern gap remains opens and Eq. (12) condition is satisfied, the SI analysis done for $|h| = 0$ uniquely determines the SIs for the $|h| \neq 0$ case. The reason for this is that the SI group for MSG #2.4, which is $\mathbb{Z}_2^3 \times \mathbb{Z}_4$, is a supergroup of $\mathbb{Z}_2^2$ in MSG #67.505. Applying the compatibility relations in MSG #67.505 to the well-know expression for the inversion SI [5–10], returns that $\{\bar{z}_{2,x}, \bar{z}_{2,y}, \bar{z}_{2,z}|\bar{z}_4\}$ can only takes value in:

$$\{0,0,0|0\}, \quad \{0,0,0|2\}, \quad \{0,0,1|0\}, \quad \{0,0,1|2\}, \tag{13}$$

thus forming $\mathbb{Z}_2^2$. The transverse SI for the photonic bands in presence of $0 < |h| << |H_z|$, computed in the MSG #2.4 setting, are:

$$\begin{aligned} \nu^{T+L}_{\phi=0} &= \{0,0,1|0\} \\ \nu^{T+L}_{\phi=\pi} &= \{0,0,1|2\} \end{aligned} \tag{14}$$

which is in agreement with Eq. (11), which were obtained in SG #13 setting. In SG #13, the $\mathcal{I}$-SI $\{\bar{z}_{2,x}, \bar{z}_{2,y}, \bar{z}_{2,z}|\bar{z}_4\}$ are reduced to

$$\bar{z}_{2,x} = \bar{z}_{2,y} = 0 \mod 2 \tag{15}$$

$$\bar{z}_{2,z} = n(\Gamma_1^-) + n(\Gamma_2^-) + n(Y_1^-) + n(Y_2^-) \mod 2 \tag{16}$$

$$\bar{z}_4 = 2n(\Gamma_2^-) + 2n(Y_2^-) + 2n(C_1^-) + 2n(Z_1^-) \mod 4 \tag{17}$$

where $n(\text{irrep})$ counts the irrep multiplicity at the corresponding HSP. There relations directly follow from applying compatibility constraints. Importantly, note that $\bar{z}_4 \in \{0,2\}$. This shows that the *relative* Axion Insulators (rAXI) with $\phi = 0,\pi$ display an obstruction of their $\bar{z}_4$ symmetry indicators, irrespective of the presence of a small $|h|$ magnetic perturbation.

II. ORIGIN-DEPENDENCE OF THE $\bar{z}_4 = 2$ INDICATOR

In this subsection, we discuss the origin-dependence of the $\bar{z}_4$ magnetic symmetry indicator for magnetic space group (MSG) #2.4 in the presence of a non-zero (and odd) background Chern contribution. We show how this fact can be leveraged to induce axion topology in photonic crystal domain walls. Consider a system in MSG #2.4 with unit cell centered at $(0,0,0)$, having the following symmetry indicators:

$$\nu_{(0,0,0)} = \{\bar{z}^0_{2,x}, \bar{z}^0_{2,y}, \bar{z}^0_{2,z}|\bar{z}^0_4\}. \tag{18}$$

When we apply a shift of the unit cell center to $(i,j,k)/2$, the new symmetry indicators can be redefined as [13]:

$$\nu_{(i,j,k)} = \{\bar{z}'_{2,x}, \bar{z}'_{2,y}, \bar{z}'_{2,z}|\bar{z}'_4\}, \tag{19}$$

where:

$$\bar{z}'_{2,x} = \bar{z}^0_{2,x} \text{mod } 2 \tag{20}$$

$$\bar{z}'_{2,y} = \bar{z}^0_{2,y} \text{mod } 2 \tag{21}$$

$$\bar{z}'_{2,z} = \bar{z}^0_{2,z} \text{mod } 2 \tag{22}$$

$$\bar{z}'_4 = \bar{z}^0_4 + 2i\bar{z}^0_{2,x} + 2j\bar{z}^0_{2,y} + 2k\bar{z}^0_{2,z} \text{mod } 4. \tag{23}$$

This shows that $\delta\bar{z}_4 = 2$ transition can be induced by a different choice of inversion center in the presence of a background odd Hall contribution (while for even Chern numbers C_l we have $\bar{z}_{2,l} = 0$). In our magnetic modulated PhCs, we have $C_x = C_y = 0$ and $C_z = 1$ which fixes $\bar{z}_{2,x} = \bar{z}_{2,y} = 0$ and $\bar{z}_{2,z} = 1$. Due to the odd C_z, we are able, with a simple shift of the supercell origin, to vary the value of the original $\bar{z}^0_4$ into $\bar{z}^0_4 + 2k$, with $k \in \mathbb{Z}$ to satisfy inversion symmetry. Note that the two phases with $\phi = 0$ and $\phi = \pi$ correspond to the cases $k = 0$ and $k = 1$, which present a relative inversion symmetry obstruction. As shown in the panels of Fig. 1(c-d) of the main text, the PhCs with $\phi = 0$ and $\phi = \pi$, are related by a translation of $(i,j,k)/2 = (1,1,1)/2$, i.e., half-lattice constant in every direction. Since the C_x, C_y Chern numbers are zero, the xy in-plane translations do not affect $\bar{z}_4$. While the absolute values of the $\bar{z}_4$ are origin-dependent for the two phases, their relative magnetic symmetry indicators are well-defined:

$$\nu_{(0,0,0)} - \nu_{(0,0,1)} = \{0,0,0|2\}, \tag{24}$$

and identify with those magnetic symmetry indicators of an axion insulator.

A. Engineering axion topology through unit cell shifts

The origin-dependence of the $\bar{z}_4$ in the presence of odd Chern numbers provides a straightforward mechanism for generating *relative* axion topology, simply by applying a relative shift across a domain wall along the direction of the Chern vector. This shift is not merely a formal change of basis but manifests in physically observable effects, such as the emergence of axionic hinge states observed in the photonic crystal domain walls. Notably, relative *rotation* of Chern vectors across domain walls has recently been demonstrated to result in the emergence of knot surface states and vectorial bulk-boundary correspondence at the interface, as originally proposed in our previous Ref. [14] and experimentally verified in Ref. [15] within photonic crystal domain walls. The present study further demonstrates that the introduction of a relative *shift* across domain walls in inversion-symmetric Chern insulators leads to the emergence of axion topology at the interface. We envision that this scheme could be applied to similar photonic crystal setups or other inversion-symmetric magnetic Hall systems with trivial *intrinsic* axion topology to induce a *relative* axion topology at their boundary. Additionally, it is possible to drive and observe a tunable axionic response by controlling the *shift* between the PhCs along their Chern vector. Beyond the practical applications associated with tunable hinge states, this approach could serve as a valuable platform to investigate the dynamic evolution of higher-order topological insulating states as they transition from the Chern to the axionic state during an adiabatic process. We plan to explore this last point in future work.

III. BOUNDARY CONDITION FOR THE DOMAIN-WALL

In the main text, we construct phase-obstructed domain walls of PhCs, with the $\phi(x)$ SM phase changing from $\phi(x) = 0$ for $x < 0$, to $\phi(x) = \pi$ for $x > 0$. Even in the presence of the domain wall, the AXI PhC is a continuous and fully connected structure, with the rods connected all across the interface. The electromagnetic modes supported by the PhC can be numerically simulated by solving the Maxwell's equations on a dense real-space grid, where discontinuities in the dielectric constant are treated via a simple linear interpolation on a dense mesh. Specifically, we use the MPB [2] in this case. Conversely, TB models are typically constructed using discrete orbitals localized at specific Wyckoff positions. To accurately capture the continuous nature of PhC geometry in the TETB model, we employ a specific boundary condition choice:

- We keep the hopping terms constant across the interface;
- We use a linear interpolation to transition between the onsite energies of the subsystems with SM $\phi = 0$ and $\phi = \pi$.

This prevents the emergence of surface effects in the TETB which deviate from the surface electromagnetic response of a fully connected 3D photonic structure. Specifically, for the model considered here, this boundary choice ensures that the SM mass term which couples the Weyl points [16]:

$$m = \Delta e^{i\phi}, \tag{25}$$

crosses zero across a domain wall where $m(x \to -\infty) = m$ and $m(x \to \infty) = -m$, where (Δ, ϕ) are amplitude and phase of the SM. This ensures the domain wall bands are gapless at criticality, as shown in Fig. S1(a).

IV. GAPLESS DIRAC CONE ON THE $x = 0$ PLANE

As shown in Fig. S1(a), the $\delta\theta = \pi$ domain wall bands display a massless 2D Dirac cone, located at $(k_y, k_z) = (\pi, \pi)$ in the surface BZ. This gapless state arises as a projection of the folded bulk Weyl points $V = (\pi, \pi, 0)$, when $\delta\theta = \pi$, and when the external magnetic field complies with the commensuration folding condition and does not present any component orthogonal to the interface plane. More specifically, since

$$m(x = 0) = 0, \tag{26}$$

the nested Weyl points reappear as a projection of the bulk Weyl points on the $x = 0$ plane, due to the local cancellation of the effects of the SM. At $x = 0$, the rAXI irreps are exchanged through the phase-obstructed domain wall as a result of the double band inversion occurring in the bulk at V, which is the point where folding of the Weyl points occurs. Importantly, this Dirac cone is not robust to perturbations at the boundary. In particular, the gapless condition for the surface modes on the $x = 0$ plane is only maintained as long as the magnetic field does not present any component orthogonal to the interface, i.e., for an x-domain wall, when

$$h_x = |h| \cos(\sigma) = 0. \tag{27}$$

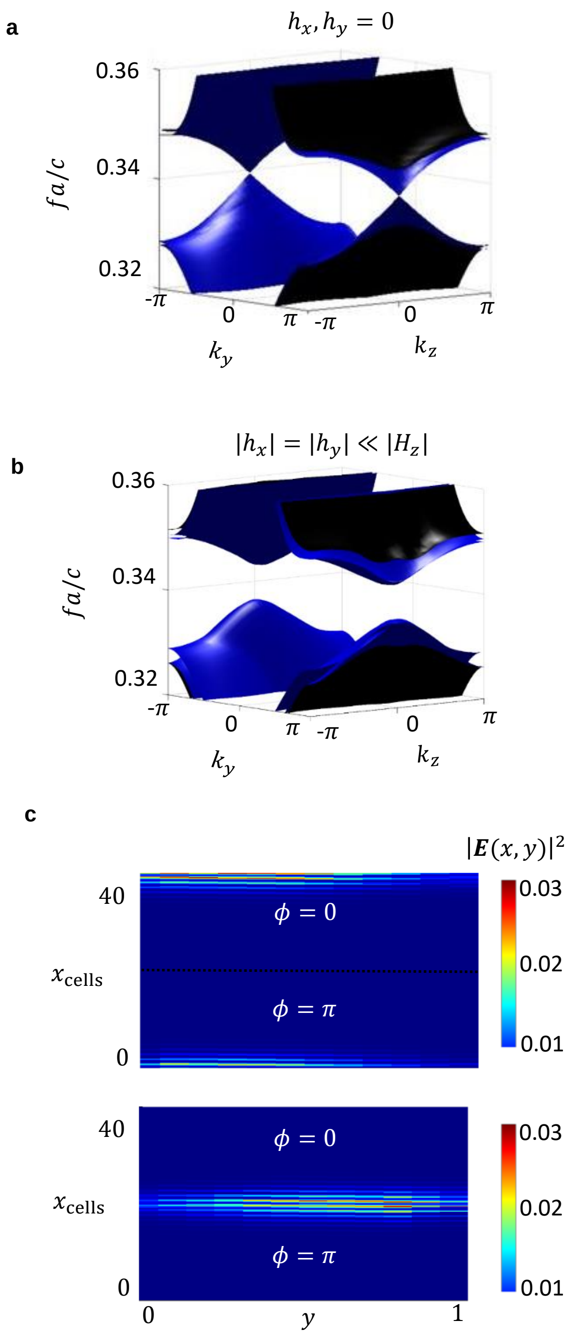

FIG. S1. Domain-wall surface bands. Case of a x domain wall with $\delta\phi = \delta\theta = \pi$. Gapless configuration in panel (a), gapped configuration in panel (b), in presence of a small magnetic perturbation $|h|$ in the xy plane. Localization of the surface modes at the interface in panel (c). Projected bulk bands in black, surface bands in blue.

V. MAGNETIC CONTROL OF THE SURFACE GAP

To impart a mass to the Dirac cone, we introduce a small magnetic perturbation in the xy plane with $|h| << |H_z|$, resulting in the gapped domain wall bands displayed in Fig. S1(b). As shown in Fig. S2, by tuning the value of $|h|$ it is possible to control the size of this surface gap. For example, for a fixed value of $\sigma = \pi/4$, the optimum gap is reached at $|h| \sim 1$. Note that $|h|$ has to be treated as a perturbation as compared to H_z: for values of $|h| \sim |H_z|$, the

gap tends to close again, due to deviation from the Weyl folding condition, and correspondent shrinking of the bulk gap.

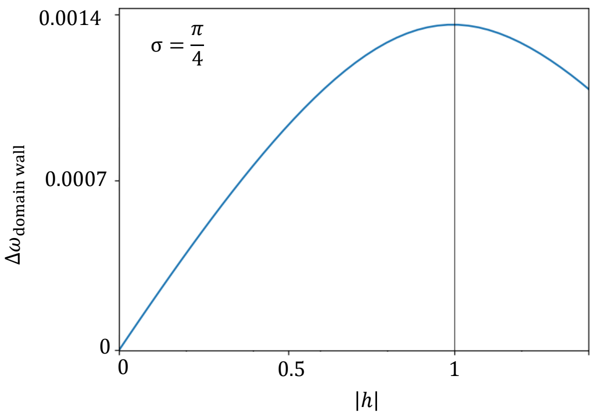

FIG. S2. Dependence of the surface gap with respect to the amplitude of the magnetic perturbation ($|h|$). Case of a x domain wall with fixed angle $\sigma = \pi/4$, as computed for the TETB model.

VI. TOPOLOGICAL TRANSITION BETWEEN DIFFERENT SURFACE HINGE CONFIGURATIONS

In the main text, we verified that it is possible to induce topological transitions across different hinge-state configurations by tuning the σ angle and rotating magnetic perturbation in the xy plane. This results in four distinct hinge-state configurations α,β,γ,δ, with localization of the upwards moving state on either of the four different hinges. Since these transitions occur across these topologically-distinct boundary configurations, we expect them to be accompanied by a gap closing point. Indeed, as shows in Fig. S3, for a x domain wall, the gap closes when the magnetic perturbation is along $\pm y$. We checked that the same happens for a y domain wall, with a gap closing when the magnetic perturbation is along 10, i.e. $\pm x$. These four values of the in-plane angle:

$$\sigma = 0, \frac{\pi}{2}, \pi, \frac{3\pi}{2} \tag{28}$$

correspond to magnetic orientations which do not break any symmetry among the four inversion-symmetric hinge-configurations.

VII. RELATIVE PHASE DIFFERENCE

As already observed, an x domain wall across phase obstructed 3D Chern photonic insulators, behaves as the critical point between AXI and a trivial insulator, as long as the SM phase satisfies $\delta\phi = \delta\theta = \pi$. Fig. S4 shows the surface bands while deviating from this condition, setting

$$-\pi < \delta\theta < \pi. \tag{29}$$

As depicted, the surface bands gradually disappear as the phase difference is tuned to 0, recovering the projected 3D Chern gap. The surface modes then reappear as the sign of the phase difference is reversed, reaching criticality again when $\delta\theta = -\pi = \pi \pmod{2\pi}$.

VIII. RELATION BETWEEN MAGNETO-ELECTRIC MEDIA AND ELECTRONIC AXIONIC INSULATORS

In what follows, we revise the concepts of axion and bi-anisotropic responses, in relation to magneto-electric effects. In particular, we highlight how these concepts are often associated, since, in condensed matter, the electromagnetic

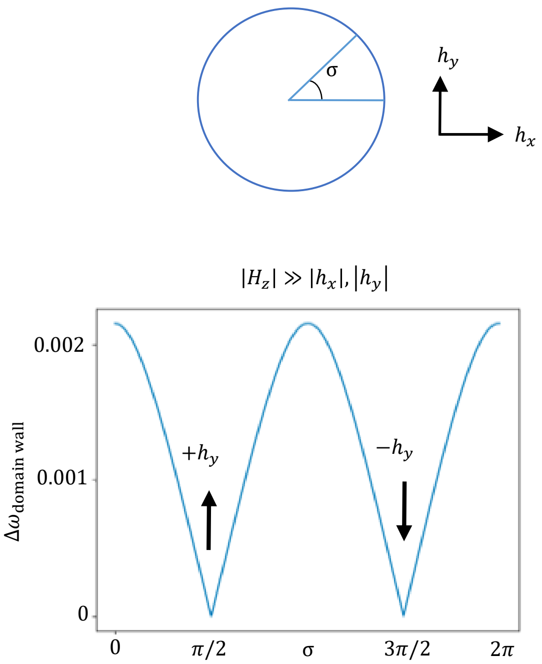

FIG. S3. Dependence of the surface gap with respect to the in-plane angle of the magnetic perturbation (σ). Case of a x domain wall with fixed amplitude $|h| = 1$, as computed for the TETB model.

response of an axion insulator parallels that of a macroscopic effective Tellegen medium.

A. Bi-anisotropic media and Tellegen responses

Bi-anisotropy, as defined in the works of Serdyukov et al. [17] and Bliokh et al. [18], refers to the magneto-electric response characterized by the terms $(\chi(\mathbf{r}), \xi(\mathbf{r}))$ that generates coupling between applied electric fields and induced magnetic fields in a medium, according to the following relations:

$$\mathbf{D}(\mathbf{r}) = \varepsilon(\mathbf{r})\mathbf{E}(\mathbf{r}) + \chi(\mathbf{r})\mathbf{H}(\mathbf{r}) \tag{30a}$$
$$\mathbf{B}(\mathbf{r}) = \xi(\mathbf{r})\mathbf{E}(\mathbf{r}) + \mu(\mathbf{r})\mathbf{H}(\mathbf{r}). \tag{30b}$$

where $\mathbf{D}(\mathbf{r})$ is the displacement field, $\mathbf{B}(\mathbf{r})$ is the magnetic flux density, $\mathbf{E}(\mathbf{r})$ is the electric field and $\mathbf{H}(\mathbf{r})$ is the magnetic field, while $\varepsilon(\mathbf{r})$ and $\mu(\mathbf{r})$ are the electric permittivy and magnetic permeability tensors. The magneto-electric coupling coefficients, $\chi(\mathbf{r})$ and $\xi(\mathbf{r})$, enter as off-diagonal terms in the 6×6 constitutive dielectric tensor, $\mathcal{K}(\mathbf{r})$:

$$\mathcal{K}(\mathbf{r}) = \begin{pmatrix} \varepsilon(\mathbf{r}) & \chi(\mathbf{r}) \\ \xi(\mathbf{r}) & \mu(\mathbf{r}) \end{pmatrix}, \tag{31}$$

which encapsulates the material's response to applied electromagnetic fields:

$$\begin{pmatrix} \mathbf{D}(\mathbf{r}) \\ \mathbf{B}(\mathbf{r}) \end{pmatrix} = \mathcal{K}(\mathbf{r}) \begin{pmatrix} \mathbf{E}(\mathbf{r}) \\ \mathbf{H}(\mathbf{r}) \end{pmatrix}. \tag{32}$$

Bi-anisotropic materials where $\chi(\mathbf{r}), \xi(\mathbf{r})$ are real-valued tensors - thus breaking time-reversal symmetry - are known as Tellegen media. The phenomenon of Tellegen response, albeit possible in natural materials, typically exhibits a negligibly small Tellegen parameter, as documented in Ref. [19]. Consequently, over recent decades, there has been

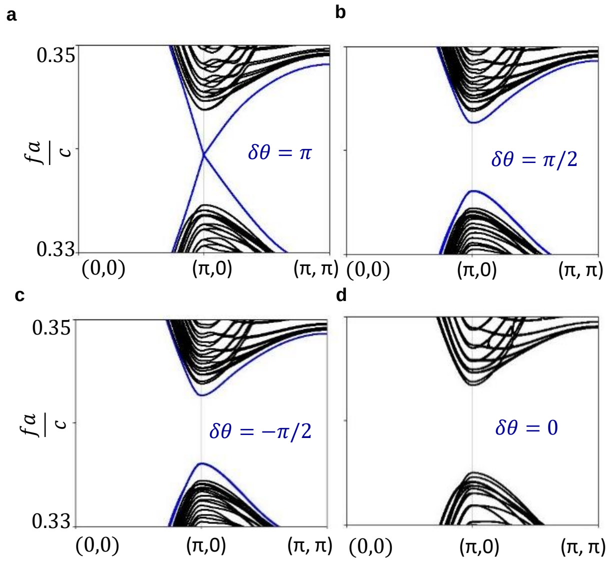

FIG. S4. Dependence of the surface gap with respect to the value of the phase difference across the interface $\delta\phi = \delta\theta$. Panels (a-c) correspond to $\delta\phi = \pi, \pi/2, -\pi/2, 0)$, respectively. Projected bulk bands in black, surface bands in blue.

a concerted effort to synthetically engineer such materials with varying degrees of success. This includes strategies like the engineering of nanoscale particles [20], metamaterials [21], and metasurfaces [22] with broken time-reversal symmetry. Dynamic approaches have also recently been proposed, such as those in [23, 24], which theoretically suggest the application of external driving and time-varying media to induce a Tellegen response.

B. Magneto-electic response of condensed matter axion insulators

As we will show, in an electronic axion insulator, a large macroscopic Tellegen response can arise solely from the static band topology encoded in the bands. The following Lagrangian term effectively describes the emergent electrodynamics of axion insulators:

$$\mathcal{L}_{\text{topo}} = -\theta \mathbf{E} \cdot \mathbf{B} \tag{33}$$

where $\theta = \theta[\mathcal{A}]$ originates from the non-trivial Berry curvature $\mathcal{A}$ of the axionic bands [25–27]. This topological term gives rise to topological quantized magneto-electric phenomena and induces a modification [28–32] in the conventional Maxwell equations:

$$\nabla \cdot (\varepsilon \mathbf{E}) = \nabla\theta \cdot \mathbf{B} \tag{34a}$$
$$\nabla \cdot \mathbf{B} = 0 \tag{34b}$$
$$\nabla \times \mathbf{E} = -\partial_t \mathbf{B} \tag{34c}$$
$$\nabla \times \mu^{-1}\mathbf{B} = \partial_t(\varepsilon \mathbf{E}) - \nabla\theta \times \mathbf{E} - (\partial_t \theta)\mathbf{B}, \tag{34d}$$

where gradients of θ act as new charges and currents, which can manifest at discontinuities and boundaries and where we have incorporated the conventional vacuum constants (ε_0, μ_0, and c^{-1}) into the definitions of the fields and the dielectric tensors . Crucially, the axion-modified Maxwell equations (Eqs. (34)) can be recast in the form:

$$\nabla \cdot \mathbf{D} = 0 \tag{35a}$$
$$\nabla \cdot \mathbf{B} = 0 \tag{35b}$$
$$\nabla \times \mathbf{E} = -\partial_t \mathbf{B} \tag{35c}$$
$$\nabla \times \mathbf{H} = \partial_t \mathbf{D} \tag{35d}$$

by considering an effective bianisotropic medium, where [29–32]:

$$\mathbf{D} = \varepsilon\mathbf{E} - \theta\mathbf{B} \tag{36a}$$

$$\mathbf{H} = \theta\mathbf{E} + \mathbf{B}/\mu. \tag{36b}$$

Given that θ, the axionic angle, is quantized and assumes real values of either 0 or π, the response of an axion insulator, in an effective medium description, can be characterized as a distinct, quantized, and macroscopic Tellegen response. Please note that the effective Tellegen response observed in the axion insulators solely *emerges from* topology encoded in the bands. This marks a departure from conventional bi-anisotropic materials since the resulting magneto-electric response is macroscopic, topological, and quantized. Importantly, it is worth noting that, as demonstrated in the present work, axion topology leads to chiral hinge modes also in photonic systems.

IX. POSSIBLE EXPERIMENTAL IMPLEMENTATION OF THE RELATIVE AXI

A. Key elements of the relative axion design

The realization of the relative axion domain wall relies on a few critical requirements and design steps:

1. Design of a time-reversal symmetry (TRS) broken Weyl semimetal:
   (a) Weyl point annihilation: Fundamental for the emergence of axion-type electrodynamics, leading to a gapped 3D Chern phase with non-zero Chern vectors.
   (b) Inversion symmetry preservation: Crucial for quantizing the axion angle.
2. Creation of a Domain Wall Configuration:
   (a) Alignment of Chern vectors: Necessary to eliminate anomalous Hall surface states.
   (b) Relative shift of the inversion-symmetry center: Essential for manifesting the relative axion topology, by creating a shift along the Chern vector.

B. Suitable experimental platforms

Gyrotropic photonic crystals (PhCs) have recently emerged as promising candidates for realizing 3D topological phases with broken time-reversal symmetry (TRS). The successful fabrication of 3D Chern insulators, resulting from Weyl point annihilation (key design feature 1.a) and maintaining inversion ($\mathcal{I}$) symmetry (key design feature 1.b), has been demonstrated in Ref. [15]. While photonic crystals with broken TRS have mainly been implemented at microwave frequencies [33, 34], recent advances in magneto-optic media hint at progress towards optical frequencies [35, 36]. The second crucial aspect of our *relative* axion design, namely the domain wall engineering (key design features 2.a and 2.b), also aligns with the latest control techniques developed for PhC domain walls [15]. Notably, the use of *relative* rotations of Chern vectors across domain walls, as explored by [15], has been employed to showcase vectorial bulk-boundary correspondence, supporting predictions made in Ref. [14]. Significantly, our approach to *relative* axion insulators at domain walls does not rely on spin-orbit coupling. This expands its potential applicability to other types of metamaterials, such as acoustic systems where topological gaps from breaking TRS [37–40], and Chern domain walls have also been explored experimentally [41].

[1] Stokes, H. T. & Hatch, D. M. Findsym: program for identifying the space-group symmetry of a crystal. *Journal of Applied Crystallography* **38**, 237–238 (2005).

[2] Johnson, S. G. & Joannopoulos, J. D. Block-iterative frequency-domain methods for maxwell's equations in a planewave basis. *Optics express* **8**, 173–190 (2001).

[3] Aroyo, M. I. *et al.* Crystallography online: Bilbao crystallographic server. *Bulg. Chem. Commun* **43**, 183–197 (2011).

[4] Elcoro, L. *et al.* Double crystallographic groups and their representations on the bilbao crystallographic server. *Journal of Applied Crystallography* **50**, 1457–1477 (2017).

[5] Watanabe, H., Po, H. C. & Vishwanath, A. Structure and topology of band structures in the 1651 magnetic space groups. *Science advances* **4**, eaat8685 (2018).

[6] Kim, H., Shiozaki, K. & Murakami, S. Glide-symmetric magnetic topological crystalline insulators with inversion symmetry. *Physical Review B* **100**, 165202 (2019).
[7] Takahashi, R., Tanaka, Y. & Murakami, S. Bulk-edge and bulk-hinge correspondence in inversion-symmetric insulators. *Physical Review Research* **2**, 013300 (2020).
[8] Xu, Y. *et al.* High-throughput calculations of magnetic topological materials. *Nature* **586**, 702–707 (2020).
[9] Elcoro, L. *et al.* Magnetic topological quantum chemistry. *Nature communications* **12**, 1–10 (2021).
[10] Po, H. C. Symmetry indicators of band topology. *Journal of Physics: Condensed Matter* **32**, 263001 (2020).
[11] Christensen, T., Po, H. C., Joannopoulos, J. D. & Soljačić, M. Location and topology of the fundamental gap in photonic crystals. *Physical Review X* **12**, 021066 (2022).
[12] Perez, A. M. *et al.* Transversality-enforced tight-binding model for 3d photonic crystals aided by topological quantum chemistry. *arXiv:2305.18257* .
[13] Song, Z., Zhang, T. & Fang, C. Diagnosis for nonmagnetic topological semimetals in the absence of spin-orbital coupling. *Physical Review X* **8**, 031069 (2018).
[14] Devescovi, C. *et al.* Vectorial bulk-boundary correspondence for 3d photonic chern insulators. *arXiv preprint arXiv:2206.04147* (2022).
[15] Liu, G.-G. *et al.* Topological chern vectors in three-dimensional photonic crystals. *Nature* **609**, 925–930 (2022).
[16] Sehayek, D., Thakurathi, M. & Burkov, A. Charge density waves in weyl semimetals. *Physical Review B* **102**, 115159 (2020).
[17] Serdyukov, A., Semchenko, I., Tretyakov, S. & Sihvola, A. Electromagnetics of bi-anisotropic materials: Theory and applications (2001).
[18] Bliokh, K. Y., Kivshar, Y. S. & Nori, F. Magnetoelectric effects in local light-matter interactions. *Physical review letters* **113**, 033601 (2014).
[19] Astrov, D. Magnetoelectric effect in chromium oxide. *Sov. Phys. JETP* **13**, 729–733 (1961).
[20] Tretyakov, S. A. *et al.* Artificial tellegen particle. *Electromagnetics* **23**, 665–680 (2003).
[21] Kodera, T., Sounas, D. L. & Caloz, C. Artificial faraday rotation using a ring metamaterial structure without static magnetic field. *Applied Physics Letters* **99** (2011).
[22] Ra'di, Y. & Grbic, A. Magnet-free nonreciprocal bianisotropic metasurfaces. *Physical Review B* **94**, 195432 (2016).
[23] Prudêncio, F. R. & Silveirinha, M. G. Synthetic axion response with space-time crystals. *Physical Review Applied* **19**, 024031 (2023).
[24] Serra, J. C. & Silveirinha, M. G. Engineering topological phases with a traveling-wave spacetime modulation. *arXiv preprint arXiv:2309.15320* (2023).
[25] Wilczek, F. Two applications of axion electrodynamics. *Physical review letters* **58**, 1799 (1987).
[26] Fu, L., Kane, C. L. & Mele, E. J. Topological insulators in three dimensions. *Physical review letters* **98**, 106803 (2007).
[27] Hughes, T. L., Prodan, E. & Bernevig, B. A. Inversion-symmetric topological insulators. *Physical Review B* **83**, 245132 (2011).
[28] Martín-Ruiz, A., Cambiaso, M. & Urrutia, L. The magnetoelectric coupling in electrodynamics. *International Journal of Modern Physics A* **34**, 1941002 (2019).
[29] Obukhov, Y. N. & Hehl, F. W. Measuring a piecewise constant axion field in classical electrodynamics. *Physics Letters A* **341**, 357–365 (2005).
[30] Karch, A. Electric-magnetic duality and topological insulators. *Physical review letters* **103**, 171601 (2009).
[31] Gratus, J., McCall, M. W. & Kinsler, P. Electromagnetism, axions, and topology: a first-order operator approach to constitutive responses provides greater freedom. *Physical Review A* **101**, 043804 (2020).
[32] Lan, Y., Wan, S. & Zhang, S.-C. Generalized quantization condition for topological insulators. *Physical Review B* **83**, 205109 (2011).
[33] Wang, Z., Chong, Y., Joannopoulos, J. D. & Soljačić, M. Reflection-free one-way edge modes in a gyromagnetic photonic crystal. *Physical review letters* **100**, 013905 (2008).
[34] Wang, Z., Chong, Y., Joannopoulos, J. D. & Soljačić, M. Observation of unidirectional backscattering-immune topological electromagnetic states. *Nature* **461**, 772–775 (2009).
[35] Onbasli, M. C. *et al.* Optical and magneto-optical behavior of cerium yttrium iron garnet thin films at wavelengths of 200–1770 nm. *Scientific reports* **6**, 23640 (2016).
[36] Luo, X., Zhou, M., Liu, J., Qiu, T. & Yu, Z. Magneto-optical metamaterials with extraordinarily strong magneto-optical effect. *Applied Physics Letters* **108** (2016).
[37] Ding, Y. *et al.* Experimental demonstration of acoustic chern insulators. *Physical Review Letters* **122**, 014302 (2019).
[38] Yang, Z. *et al.* Topological acoustics. *Physical review letters* **114**, 114301 (2015).
[39] Ni, X. *et al.* Topologically protected one-way edge mode in networks of acoustic resonators with circulating air flow. *New Journal of Physics* **17**, 053016 (2015).
[40] Khanikaev, A. B., Fleury, R., Mousavi, S. H. & Alu, A. Topologically robust sound propagation in an angular-momentum-biased graphene-like resonator lattice. *Nature communications* **6**, 8260 (2015).
[41] Linyun, Y. *et al.* Acoustic three-dimensional chern insulators with arbitrary chern vectors. *arXiv preprint arXiv:2401.07040* (2024).
